# Network analysis of England's single parent household COVID-19 control policy impact: a proof-of-concept study

**DOI:** 10.1017/S0950268822000905

**Published:** 2022-05-16

**Authors:** N. L. Edelman, P. Simon, J. A. Cassell, I. Z. Kiss

**Affiliations:** 1 School of Sport and Health Sciences, University of Brighton, Brighton, UK; 2 Primary Care & Public Health, Brighton & Sussex Medical School, Brighton, UK; 3 Department of Applied Analysis and Computational Mathematics, Institute of Mathematics, Budapest, Hungary; 4 Department of Mathematics, School of Mathematical and Physical Sciences, University of Sussex, Falmer, Brighton BN1 9QH, UK

**Keywords:** COVID-19, epidemiology, health policy, transmission

## Abstract

Lockdowns have been a core infection control measure in many countries during the coronavirus disease 2019 (COVID-19) pandemic. In England's first lockdown, children of single parent households (SPHs) were permitted to move between parental homes. By the second lockdown, SPH support bubbles between households were also permitted, enabling larger within-household networks. We investigated the combined impact of these approaches on household transmission dynamics, to inform policymaking for control and support mechanisms in a respiratory pandemic context. This network modelling study applied percolation theory to a base model of SPHs constructed using population survey estimates of SPH family size. To explore putative impact, varying estimates were applied regarding extent of bubbling and proportion of different-parentage within SPHs (DSPHs) (in which children do not share both the same parents). Results indicate that the formation of giant components (in which COVID-19 household transmission accelerates) are more contingent on DSPHs than on formation of bubbles between SPHs, and that bubbling with another SPH will accelerate giant component formation where one or both are DSPHs. Public health guidance should include supportive measures that mitigate the increased transmission risk afforded by support bubbling among DSPHs. Future network, mathematical and epidemiological studies should examine both independent and combined impact of policies.

## Introduction

The coronavirus disease 2019 (COVID-19) pandemic has had a devastating impact around the world, with politicians implementing population infectious disease control measures including ‘lockdowns’, defined by the Cambridge dictionary as: ‘a period of time in which people are not allowed to leave their homes or travel freely, because of a dangerous disease’.

After reporting its first case in January 2020, the UK experienced an early and large spike in excess deaths (7.7% above the 5-year average by the end of that year) [1]. The first national lockdown in England was imposed on 23rd March 2020, and eased through June/July of that year. The ‘stay at home’ rules exempted children from ‘single parent households’ (SPHs) who routinely stayed with or visited both parents, allowing them to continue to do so. After a flattening and decline in the epidemic curve through the summer a second English lockdown was imposed on 3rd November 2020 and re-introduced on 6th January 2021 after being briefly lifted through December using a tiered system of restrictions. Other social-distancing measures implemented to varying degrees across England, the UK and other countries include disallowing and/or limiting individuals being inside others' homes.

Following the first lockdown evidence emerged of the negative impact of social distancing on mental well-being [2]. Social support was found to have an important protective effect against this [3]. Therefore, from September 2020 some households in England were permitted to form a ‘support bubble’ with another household [4]. SPHs were among these designated household types, defined as ‘a single adult living with one or more children who are under the age of 18 or were under that age on 12 June 2020’ [4]. The Office for National Statistics (ONS) estimates there were 2.9 million lone parent families (LPFs) in the UK in 2020 (14.7% of all families). This calculation attributes each child to *one* household based on which parent receives the child benefit (a payment available to all families up to income £50 000 for the highest earner). In 86% of LPFs the child(ren) resides predominantly with the mother [5]. Estimates of LPF size differ greatly. ONS Families and Household data for 2018 found that 55% of LPFs had one child, 32% had two and 13% comprised of three or more (<18 years of age); while 2020 data found 30.17% had one child, 37.34% had two and 32.49% had three or more. Among LPFs there is no available data on different-parentage (in which the children of that household do not share all the same parents/caregivers), although US data suggest that 28% of all women with two or more children had those children by more than one father [6].

The risk of within-household transmission of severe acute respiratory syndrome coronavirus 2 (SARS-CoV-2) is estimated at 10 times greater than transmission through non-household contacts [7], and is thought to account for 70% of transmission [8]. The household secondary attack rate (SAR) (the percentage of contacts of an index case who become infected) has been estimated at 16.6% – varying by age, relationship type, presence of symptoms and number of household members [9]. Network analysis has aided understanding of transmission dynamics where household living arrangements are combined with movement of actors between homes; a recent study of domiciliary care demonstrating the impact on COVID-19 transmission of that movement [10].

Infectious disease transmission among individuals connected via a contact network can be mapped using percolation theory [11, 12]. Put simply, the transmission of an epidemic between network nodes requires ‘activation’ of links along which infection has spread. Keeping such links and discarding all other, also referred to as ‘bond percolation’, results in a network with fewer links, since not all existing links will transmit. The severity of an epidemic is directly related to the size of connected components (i.e. a subset of nodes where any two nodes are connected in both directions) in this sparser network. A ‘giant component’ is a subset of nodes (e.g. adults and children in SPHs) such that any two nodes (people) in this subset can be connected using the available links (such as those afforded from offspring moving between parental homes and/or support bubbling), and where the number of nodes in this subset scale with the size of the full network. Where a giant component is formed, a large epidemic is likely as the infection can ‘percolate’ through the network. Conversely, if the sparser network comprises many disconnected components, the likelihood of a large epidemic is small. In percolation theory, one is interested in the point at which the extent of connectivity between people leads to the formation of a giant component. From a public health perspective, this is particularly valuable in identifying the critical percolation threshold at which an infection (such as SARS-CoV-2) is likely to affect a large proportion of individuals. Percolation theory has already been applied to investigate putative impact on COVID-19 transmission of forming household bubbles, finding that reduced contact outside the bubble mitigated the transmission impact of bubbles with more than two members [13]. Network analysis has also explored the potential impact on COVID-19 transmission of ‘contact clustering’ in social bubbles (as part of an imagined exit strategy), similarly finding negligible impact where those bubbles remained contained [14]. However, both studies assume exclusivity between bubbling households, and do not account for domiciliary movement between households, for example through care workers or the children of SPHs. To our knowledge, no studies have investigated the cumulative impact on COVID-19 transmission of SPH support bubbles in the context of SPH child movement between parental residences.

**Fig. 1.**
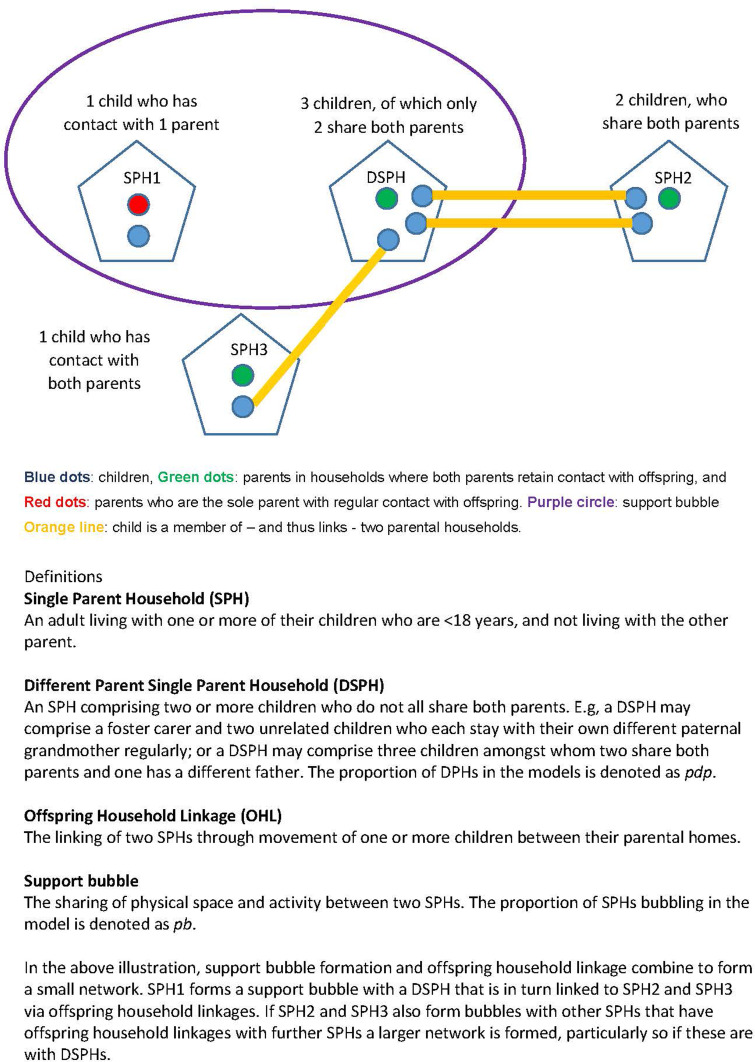
Illustration of terms. Blue dots: children, green dots: parents in households where both parents retain contact with offspring and red dots: parents who are the sole parent with regular contact with offspring. Purple circle: support bubble: child is a member of – and thus links – two parental households. Definitions: *Single parent household (SPH):* An adult living with one or more of their children who are <18 years, and not living with the other parent. *Different parent single parent household (DSPH):* An SPH comprising two or more children who do not all share both parents. E.g. a DSPH may comprise a foster carer and two unrelated children who each stay with their own different paternal grandmother regularly; or a DSPH may comprise three children among whom two share both parents and one has a different father. The proportion of DSPHs in the models is denoted as *p_dp_*. *Offspring household linkage (OHL):* The linking of two SPHs through movement of one or more children between their parental homes. *Support bubble:* The sharing of physical space and activity between two SPHs. The proportion of SPHs bubbling in the model is denoted as *p_b_*. In the above illustration, support bubble formation and OHL combine to form a small network. SPH1 forms a support bubble with a DSPH that is in turn linked to SPH2 and SPH3 via OHLs. If SPH2 and SPH3 also form bubbles with other SPHs that have OHLs with further SPHs a larger network is formed, particularly so if these are with DSPHs.

This proof-of-concept study aimed to examine the impact of two mechanisms through which a network of SPHs in which children are spending time with parents who live in different households can become more connected, leading to larger giant components with a higher probability of a large outbreak. Here, we use the term ‘parent’ to include all primary care givers regularly resident in the same household with the child. The first mechanism was the extent of different-parentage within SPHs (DSPHs). DSPHs comprise two or more children who only share one parent and have different parents in other SPHs with whom they also stay with regularly. As an example, a DSPH might comprise a foster carer, child A and child B who are unrelated and each stay with their own paternal grandmother regularly. The second mechanism was the extent of bubble formation between one SPH and another SPH which does not include the other parent of any of the offspring. For example, SPH1 may bubble with SPH2 while the two offspring of SPH1 each have a parent they stay with regularly who reside in SPH3 and SPH4 respectively rather than in SPH2 with whom the bubble is formed. Figure 1 depicts these associations graphically.

We hypothesise that this combination of SPH policies might contribute to the development of networks that could facilitate COVID-19 transmission. This focus on network formation can offer insights that can contribute towards our understanding of COVID-19 household transmission rates. In turn, the evidence generated may inform future decisions about the need for, and targeting of, support and infection control measures concerning contact between households in the context of COVID-19 and other respiratory pathogens.

## Methods

**Fig. 2.**
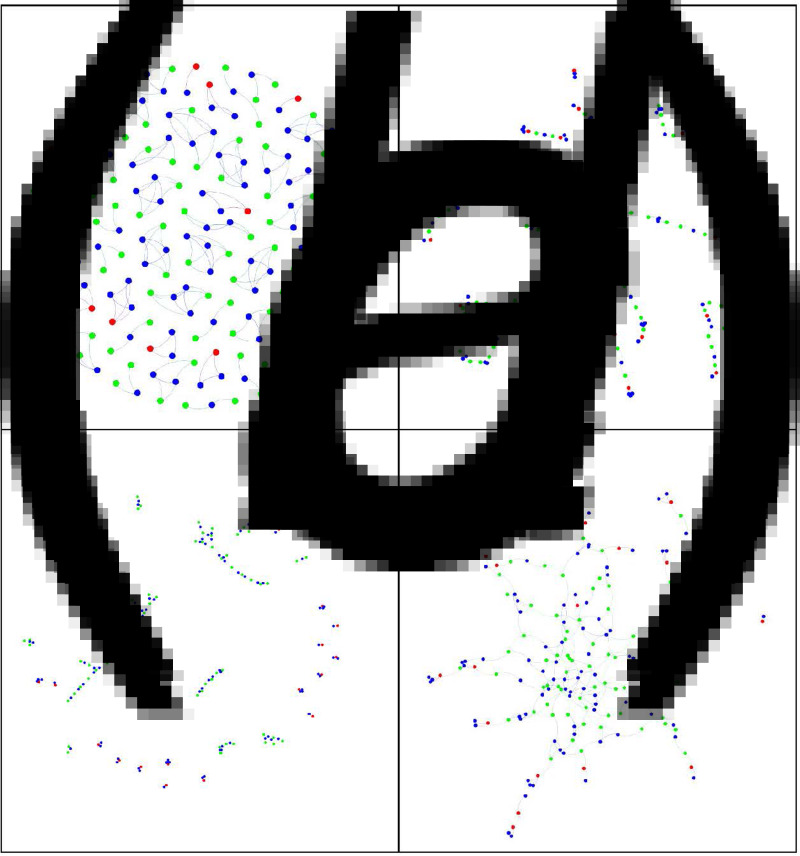
Plotted networks. Plots of ‘toy’ contact networks (representing a reduced number of nodes for visual illustration of *N* = 186) with different levels of DSPHs and bubbling. (A) Baseline model without any discordant-parentage and without any bubbling, (B) bubbling only, (C) discordant-parentage only and (D) both discordant-parentage and bubbling. Nodes are colour code as follows: blue dots – children, green dots – parents in households where both parents retain contact with offspring, and red dots – parents who are the sole parent having regular contact with their offspring.

For the purposes of this study we defined a single parent as a parent (under the definition above) who does not live with the other primary caregiver of their child or children, irrespective of whether they are living with a new partner or not. We therefore defined an SPH as a household in which a single parent (using our definition) resides with one or more of children at least 10% of the time. Thus, a child who stays with each parent at least one night in every fortnight is considered a member of two SPHs. This can be seen in panel A of Figure 2 where for example a child (blue dot) is connected to two parents (green dots), where the parents themselves are not connected. As the study aimed to produce a static model we henceforth use the term ‘offspring household linkage’ (OHL) to denote the movement of children between the two SPHs in which their parents' reside. We hypothesised that the combined effects of SPHs bubbling with each other *in addition to* OHL of SPHs outside of those bubbles (i.e. children alternating time with each parent) would contribute to COVID-19 household transmission.

We further hypothesised that where an SPH is a DSPH (comprising two or more children who have parents in other SPHs with whom they also stay with regularly as described above), this would additionally contribute to network transmission and the formation of a giant component. We defined households of this type as different-parentage single parent households. These definitions of SPH, DSPH and OHL support the analysis of COVID-19 transmission by placing focus on children spending time with parents in different SPHs and on eligibility for support bubble formation, rather than on legal residency of the child or current relationship status of the parent. In this study, we developed a simple mechanistic network model upon which to explore the impact of DSPHs and bubbling as mechanisms that can increase the connectivity in the network and thus the risk of larger outbreaks.

Relevant parameters for base model construction were drawn from population survey estimates, where available. UK data on rates of parent contact among dependent children of separated or divorced parents are sparse. A 2013 survey of non-resident fathers found that 13% reported no contact with their child with 59% reporting contact at least once a week; this survey did not specify whether that contact was face-to-face but 49% reported their children staying with them on a weekly basis [15]. An earlier 2007 study using ONS data found that approximately 35% children in SPHs stayed with both parents on a weekly basis with a further 25% staying less than once a week but more than once a month [16]. We used these data as context, developing our model with the assumption that 80% of children would spend time at both their parents' SPHs once a fortnight or more frequently. We made this assumption on the basis of the above ONS data indicating 60% of SPH children stayed with both parents more than once a month with a further 20% assumed likely to have face-to-face contact with both parents even if not staying overnight.

Given the varied reports of SPH size (as reported in the Introduction) and the lack of direct comparability between the ONS definition of LPFs and our own definition of SPHs we used parameter estimates representing mid-points such that 42% of SPHs included one child, 35% had two children and 23% had three or more.

We therefore defined a base model such that the number of SPHs with one, two and three children is 420, 350 and 230, respectively. To account for circumstances where contact between one parent and their offspring has ceased, the model was constructed such that 20% of all SPHs did not have an OHL to another SPH. Such households are visible in panel A of Figure 2 where parents are denoted by red dots and all children are represented by blue dots. These are in line with the figures reported above. These offspring linkages (i.e. the connections between SPHs that arise from children staying in each parent's SPH at least once a fortnight) are built into our base model – i.e. we did not model removal of movement between parental homes on the basis that this would not be an ethically acceptable component of any lockdown in the UK context.

The network is constructed by first focusing on the 80% of the SPHs. Knowing these numbers, say *N*_*SPH*1_, *N*_*SPH*2_ and *N*_*SPH*3_, determine the number of children in the network at this stage, that is *N*_*CH*_ = (*N*_*SPH*1_ + 2 × *N*_*SPH*2_ + 3 × *N*_*SPH*3_)/2. A proportion of *r*_1_, *r*_2_ and 1 − (*r*_1_ + *r*_2_) of the children are then allocated to SPHs with one, two and three children, respectively. This is done proportionally to the number of stubs starting from SPHs, that is 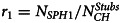, 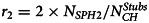 and 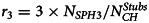, where 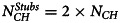. This means that for example, *r*_1_ × *N*_*CH*_ children will be allocated to SPHs with one child, and the same approach is applied to SPHs with two and three children. At this point, all children will have exactly one spare stub remaining, that is the stub needing a second parent. Equally, there will be parents with no children allocated to them at this point. This leaves us with a good degree of flexibility to vary the DSPHs. For example, one can impose that a stub from a child already allocated to an SPH connects to a parent with one single stub, i.e. to a parent who is in the pool of SPHs with one child. Conversely, the spare stub from this same child could be connected to a spare stub from a parent in the pool of SPHs with two or three children. The degree to which DSPHs is enforced is captured by the probability *p*_*dp*_, where a value of zero means that all spare stubs from children in SPHs with one, two or three children are allocated to parents that are in the pool of SPHs with one, two or three children, respectively. When this probability is close to one, the degree of DSPHs is pushed to maximum within the constraints of being able to construct the network.

This process is followed by supplementing the network with an extra 20% of SPHs (for all three types of SPHs) with one parent only. These are isolated fully connected ‘cliques’ comprising two, three or four nodes, respectively (see Fig. 2, panel A) with parents (red dots) and children (blue dots). Such households do not contribute to the extent of DSPHs.

Next, we form bubbles between pairs of parents that do not share offspring. All permissible pairs are chosen at random and connected with probability *p*_*b*_, i.e. the probability of bubble formation across a given pair.

Once the network model is implemented, we vary (*p*_*dp*_, *p*_*b*_) and measure the size of the giant component across many realisations. We also measure the average degree in the network and consider the distribution of connected components to understand how the giant component emerges.

## Results

First, we demonstrate the effect of the two mechanisms by explicitly plotting the contact network for networks of relatively small size, in this case *N* = 186 individuals.

**Fig. 3.**
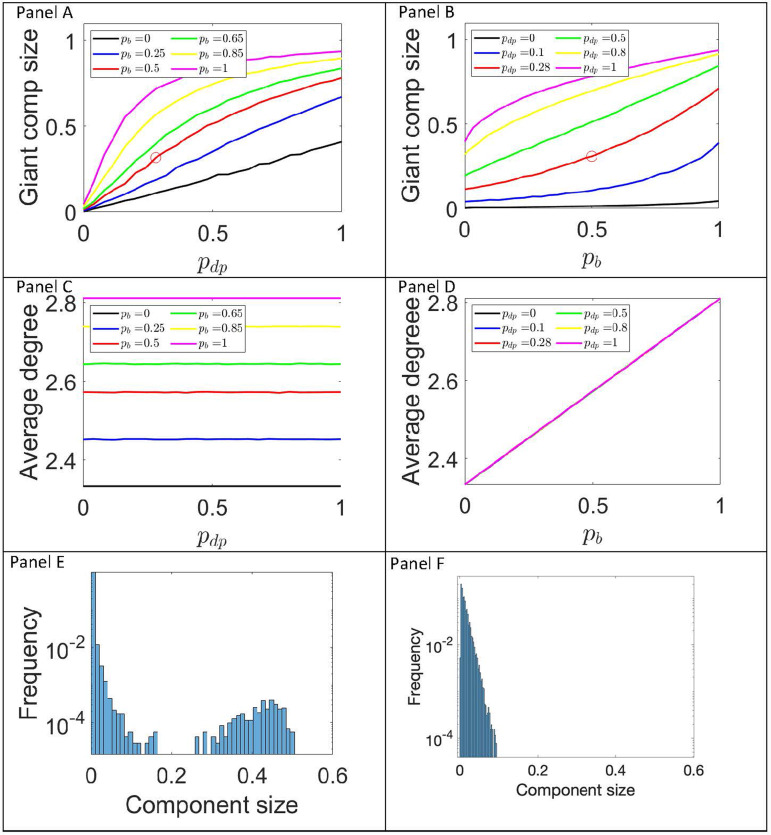
Incremental impact of DSPHs and bubbling on giant component formation. Plots of the giant component size (top row), average degree (middle row) and distribution of connected component sizes (bottom row) as a function of increasing levels of DSPHs (left column) and bubbling (right column) based on a network model of *N* = 2086 nodes. The last row gives the distribution of component size as a proportion of total population size for (p_dp_, p_b_) = (1, 0) (panel E) and (p_dp_, p_b_) = (0, 1) (panel F) based on averaging 250 simulations. The red circles in the top row correspond to (p_dp_, p_b_) = (0.28, 0.5), values that have been observed in practice.

Figure 2, panel A depicts the baseline model. The network consists of isolated clusters where children (blue dots) are connected to siblings and to one (red nodes) or two (green nodes) parents. Figure 2, panel B illustrates bubbling alone, and panel C connectivity from DSPHs alone. The greatest connectivity occurs where DSPHs and bubbling are combined (Fig. 2, panel D). Bubbling alone forms multiple chains of smaller sizes (panel B) from which emergence of a large chain is unlikely. However, as different-parentage becomes more common, the opportunities for linkage creation between SPHs increase. For example, three children in an SPH with their father may have three different mothers, each of whom is a part of another SPH, creating more chances to connect up isolated chains of SPHs. These trends are clearer in the simulations performed on larger networks (Fig. 3).

We now move on to report on results for these larger networks of *N* = 2086 nodes (this seemingly arbitrary number arises as SPHs with one, two and three children and their numbers lead to some conditions that are necessary to construct the network). We studied the full spectrum of (*p*_*dp*_, *p*_*b*_) values, with special focus on (*p*_*dp*_, *p*_*b*_) = (0.28, 0.5) which seems to be close to some of the estimates in the literature described above. These demonstrate how several networks were created with a given parameter set and the measured values averaged across these realisations.

The plots in Figure 3 demonstrate the incremental impact of the two mechanisms by which the contact network can lead to the formation of a larger giant component and thus a higher probability of a large outbreak. Panels A and C depict the probability of SPHs of two or more children comprising DSPHs, described by the value *p*_*dp*_, and assessing the impact of this on network connection for various fixed probabilities of bubble formation (*p*_*b*_) (depicted with different coloured lines). Panels B and D depict the probability of SPHs forming bubbles with other SPHs (that do not include the other parent of that SPH's offspring), described by the value (*p*_*b*_) and assessing the impact of this on network connections for various fixed probabilities of DSPHs (*p*_*dp*_) (depicted again with coloured lines).

The top row of Figure 3 illustrates that the growth of the giant component is much slower for bubbling without DSPHs than for DSPHs without bubbling. DSPHs create bigger components such that addition of bubbling events allows for giant components to emerge. This is clearly visible if we inspect the black curves in panels A and B of Figure 3. It appears that bubbling by non-DSPHs forms a giant component only very slowly, with many smaller components emerging that nonetheless do not easily percolate into a single giant component. However, as *p*_*dp*_ increase, and with no bubbling, the giant component grows faster. This is despite the average number of links staying constant under various values of *p*_*dp*_ and increasing as *p*_*b*_ increases, see middle row of Figure 3. The intuition behind this is provided by looking at the bottom row of Figure 3. It is clear that as *p*_*dp*_ approaches its maximum value a giant component can emerge. This is not the case for value of *p*_*b*_ close to 1, where all components remain of small size.

Figure 3 includes a point estimate (see red circles) that represents the most likely England scenario regarding prevalence of DSPHs and of bubbling. This point estimate assumes that 28% of SPHs comprise two children or more are DSPHs (based on US data and making the conservative assumption in the case of three or more children each child would not have a different other parent), and that 50% of SPHs would form a support bubble. The growth of the giant component is faster as the level of DSPHs increases compared to when bubbling increases. Furthermore, the lines in panel C correspond to networks with the same average degree. On the one hand, this is because the level of DSPHs does not change the number of links in the network; it simply re-distributes links. However, when bubbling is acting then the network gains more links. On the other hand, this means that the same number of links can be distributed in order to increase or decrease the size of the giant component. In panel D, it is evident that fixing *p*_*b*_ = 0.5 leads to an average degree of about 2.55 but with giant component sizes ranging from 0.1 to 0.8 approximately.

Finally, formation of the giant component under different mechanisms (different parentage and bubbling) is shown in Figure 2. Panel B in Figure 2 shows that bubbling leads to networks where nodes in the giant component are connected in a linear fashion, meaning that the path length within the giant component is close to the actual size of the giant component. In panel D of the same figure, we see that increasing the prevalence of different parentage leads to giant components which are better connected, where path length within the giant component is less than that in the case of bubbling. In the percolation setting, paths correspond to the infection transmission so they are related to the timescale of the epidemic. A giant component that is more densely connected will lead to an epidemic that grows faster when compared to a giant component where nodes are connected in a linear fashion as explained above. Hence, the implications are that under bubbling alone the epidemics will be of longer duration and at low prevalence.

## Discussion

This proof-of-concept study demonstrates that support bubbles between SPHs generally have little impact on formation of giant components that may cause COVID-19 outbreaks, except where one or more are DSPH. This is because OHLs from DSPHs have a greater impact on giant component formation than support bubbling with another SPH. The cumulative effect of DSPHs forming bubbles with other SPHs or DSPHs likely speeds up the formation of giant components through which COVID-19 transmission would occur.

This is the first study to model the combined effects of two SPH-related COVID-19 infection control measures by examining the added impact of bubble formation *between* SPHs against a backdrop of SPH network linkage created by movement of offspring between parental homes. It used prevalence estimates of SPH number of children and rates of contact with both parents for children of SPHs. In the absence of good estimates for rates of different-parentage and extent of bubbling between SPHs, the study design allowed exploration of the variable impact of each on giant component formation – modelling separate and combined impacts.

## Limitations

This study modelled connectivity between individuals in SPHs, rather than COVID-19 transmission itself, so that the absolute impact of public health policy on transmission cannot be evaluated and was not the purpose of the study. The exact contribution of children to COVID-19 transmission will vary over time according to deployment of infection control strategies, epidemic size and stage, vaccination programmes and the dominance of different variants. Nonetheless, two studies have suggested that the SAR within-households from child to adult is much lower than that from adult to child [17, 18], although the asymptomatic nature of paediatric infections and exclusion of adult–child pairs who shared a common initial exposure may have led to underestimation.

As a proof-of-concept study, this work also did not take account of *all* variables likely to affect household transmission and worked under certain hypotheses concerning family size and constitution. For example, distribution of offspring age was not accounted for, although younger children are less likely to transmit COVID-19 [19] while older children are more likely to lose contact with their fathers, which would reduce OHL. In the absence of available data, we were not able to account for variation in the amount of time that a child spent with each parent, or the frequency of movement between households.

In the interest of simplicity, our models did not account for the additional effect of childcare bubbling (open to all households with children aged 14 years or younger, but with close contact minimised). The prevalence of this is not well documented and likely to have variable impact on transmission depending on the extent of contact; Public Health England guidance advised that an SPH could meet simultaneously with its childcare bubble and support bubble. As this study focused on SPH connectivity rather than SPH-related COVID-19 transmission itself, the models did not account for school-related infection control measures. When schools are open the differential impact of DSPHs forming support bubbles may well be negligible, although there is conflicting evidence for the impact of school closures on transmission [18, 20].

Use of real-life data to test our hypothesis and validate the models is not currently possible, as surveillance has not captured uptake and type of support bubbles. Nor do population surveys adequately capture the size and composition of SPHs and movement of offspring between them. Surveillance data during health emergencies needs to better capture uptake of policies to inform decision-making. This study demonstrates the importance of scientists and policy makers considering the potential impact of not only *individual* infection control measures, but of their potential *combined* effects too. This supports not only accurate assessment of *overall* impact, but also identification of differential impacts that require mitigations to better support specific sub-populations. The findings indicate that support bubbles generally make little contribution to COVID-19 transmission. However, potential for significant contribution to transmission is greater with bubbling involving DSPHs.

Support bubbles are an important strategy for social and psychological support when social interaction is restricted; the findings suggest that additional support strategies may be needed to mitigate increased risk of transmission from support bubbling among DSPHs. Importantly, low income is associated with dense living accommodation [21], and also with both parental separation and higher numbers of children for women, who are more often the primary carer [22]. Lower income has also been found to be associated with reluctance to test for COVID-19 with significantly lower testing rates in areas of economic deprivation, widely attributed to the economic impact of self-isolation where individuals are on zero-hour contracts or in other unstable employment [23]. The increased potential for transmission where DSPHs bubble, also points to the need for effective mitigations against household transmission – such as ventilation – that recognise the structural vulnerability of these families. Strategies to mitigate potential increased risk among DSPHs might include frequent testing, co-produced health promotion messaging and adequate financial recompense for those self- isolating – aimed more generally at populations among whom transmission risks are higher for these socio-economic and structural reasons. More generally, awareness of potential giant component formation should underpin public health policy with regards to individuals more vulnerable to COVID-19 acquisition, including those interacting in complex multi-household structures.

## Data Availability

The data that support the findings are available by direct communication with Professor Istvan Kiss at I.Z.Kiss@sussex.ac.uk in line with University of Sussex policy.
